# Off-Target Effects of Psychoactive Drugs Revealed by Genome-Wide Assays in Yeast

**DOI:** 10.1371/journal.pgen.1000151

**Published:** 2008-08-08

**Authors:** Elke Ericson, Marinella Gebbia, Lawrence E. Heisler, Jan Wildenhain, Mike Tyers, Guri Giaever, Corey Nislow

**Affiliations:** 1 Department of Pharmaceutical Sciences, University of Toronto, Toronto, Ontario, Canada; 2 Department of Molecular Genetics, University of Toronto, Toronto, Ontario, Canada; 3 School of Biological Sciences, The University of Edinburgh, Edinburgh, United Kingdom; 4 Samuel Lunenfeld Research Institute, Mount Sinai Hospital, Toronto, Ontario, Canada; 5 Banting and Best Department of Medical Research, University of Toronto, Toronto, Ontario, Canada; Yale University, United States of America

## Abstract

To better understand off-target effects of widely prescribed psychoactive drugs, we performed a comprehensive series of chemogenomic screens using the budding yeast *Saccharomyces cerevisiae* as a model system. Because the known human targets of these drugs do not exist in yeast, we could employ the yeast gene deletion collections and parallel fitness profiling to explore potential off-target effects in a genome-wide manner. Among 214 tested, documented psychoactive drugs, we identified 81 compounds that inhibited wild-type yeast growth and were thus selected for genome-wide fitness profiling. Many of these drugs had a propensity to affect multiple cellular functions. The sensitivity profiles of half of the analyzed drugs were enriched for core cellular processes such as secretion, protein folding, RNA processing, and chromatin structure. Interestingly, fluoxetine (Prozac) interfered with establishment of cell polarity, cyproheptadine (Periactin) targeted essential genes with chromatin-remodeling roles, while paroxetine (Paxil) interfered with essential RNA metabolism genes, suggesting potential secondary drug targets. We also found that the more recently developed atypical antipsychotic clozapine (Clozaril) had no fewer off-target effects in yeast than the typical antipsychotics haloperidol (Haldol) and pimozide (Orap). Our results suggest that model organism pharmacogenetic studies provide a rational foundation for understanding the off-target effects of clinically important psychoactive agents and suggest a rational means both for devising compound derivatives with fewer side effects and for tailoring drug treatment to individual patient genotypes.

## Author Summary

Neuropsychiatric disorders such as depression and psychosis affect one-quarter of all individuals during their lifetime, and despite efforts to improve the selectivity of psychoactive drugs, all are associated with side effects. Drug efficacy and tolerance are known to be linked to an individual's genetic profile, but little is known about the nature of this correlation due, in part, to the current emphasis on screening compounds against targets *in vitro*. Here we present a comprehensive, genome-wide effort to understand drug effects on the cellular level using an unbiased genome-wide assay to determine the importance of every yeast gene for tolerance to 81 psychoactive drugs. We found that these medications perturbed many evolutionarily conserved genes and cellular pathways, such as those required for vesicle transport, establishment of cell polarity, and chromosome biology. The 500,000 drug–gene measurements obtained in this study increase our understanding of the mechanism of action of psychoactive drugs. Specifically, this study provides a framework to assess the next generation of psychoactive agents and to guide personalized medicine approaches that associate genotype and phenotype.

## Introduction

Neuropsychiatric disorders will effect 25% of all individuals at some point in their lives, with devastating social and economic consequences [1]. This constellation of diseases encompasses schizophrenia, depression, age-related memory and cognition decline, and the degeneration of neuromuscular function. Most prescribed psychoactive drugs are thought to primarily target neurotransmission pathways in the central nervous system, and thereby cause changes in perception, mood, consciousness, and behavior. Many of these therapeutics have been developed using *in vitro* assays and, as such, may have other unknown targets and unanticipated cellular effects *in vivo*. For example, side effects of antipsychotic drugs include tremors, hypotension, impotence, lethargy, and seizures [2]. In an effort to improve efficacy and to reduce side effects, new generations of drugs have been developed; among these are the so-called atypical antipsychotics such as clozapine. While clozapine is linked to a reduced risk of neuromuscular side effects, it is associated with new side effects such as life-threatening agranulocytosis in up to 1% of patients [3], and, less frequently, fatal myocarditis [4]–[6]. As such, the therapeutic benefit of this and other new atypical drugs remains open to debate. For example, a comprehensive meta-regression analysis that compared both typical and atypical drugs concluded that atypical antipsychotics were neither more effective nor better tolerated than conventional agents [7]. Other classes of psychoactive drugs, such as the antidepressants, also cause numerous undesirable side effects and the broad usage of these medications have been questioned [8].

Surrogate genetics is an effective approach to interrogate heterologous gene function or drug mechanism of action using simpler model organisms [9],[10]. The budding yeast *Saccharomyces cerevisae* has previously been used to help elucidate the basis of some psychiatric disorders [11]–[18]. For example, the expression in yeast of mutant and wildtype forms of the Huntington's disease gene revealed important factors regulating the toxicity of protein aggregates [11],[15],[19], and a genome-wide suppressor screen in yeast uncovered kynurenine 3-monooxygenase as a potential new therapeutic target for the treatment of Huntington's disease [13]. In other studies, expression in yeast of the alpha-synclein gene associated with Parkinson's disease yielded a network of interacting genes that modulate cellular toxicity [11],[15],[19].

Recently, the genome-wide collection of yeast gene deletion strains has been used to generate genetic profiles of drug sensitivity and resistance [20]–[26]. These profiles have uncovered unexpected mechanisms of action for well-known drugs, such as for the anti-metabolite 5-fluorouracil in perturbation of rRNA processing [22],[25] and for the anti-cancer agent tamoxifen in calcium homeostasis [26].

To better understand potential off-target effects of FDA-approved psychoactive drugs and their analogs, we profiled 214 psychoactive compounds in quantitative wildtype yeast growth assays and generated genome-wide deletion sensitivity profiles for the 81 drugs that caused overt growth defects. The sensitivity profiles for 49 of these drugs were overrepresented for core cellular functions such as chromatin organization, establishment of cell polarity, and membrane organization and biogenesis. Our results provide a rational foundation for personalized drug approaches and for understanding unwanted side effects in clinically important psychoactive agents.

## Results

### Specific Classes of Psychoactive Drugs Have Bioactivity in Yeast

**Figure 1:**
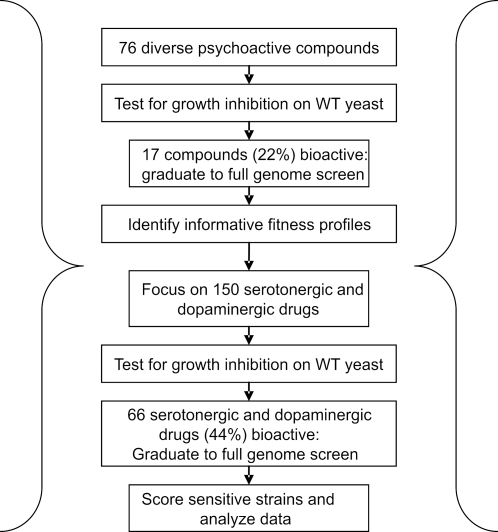
Workflow. Schematic overview of the chemical genetic screening process.

**Figure 2:**
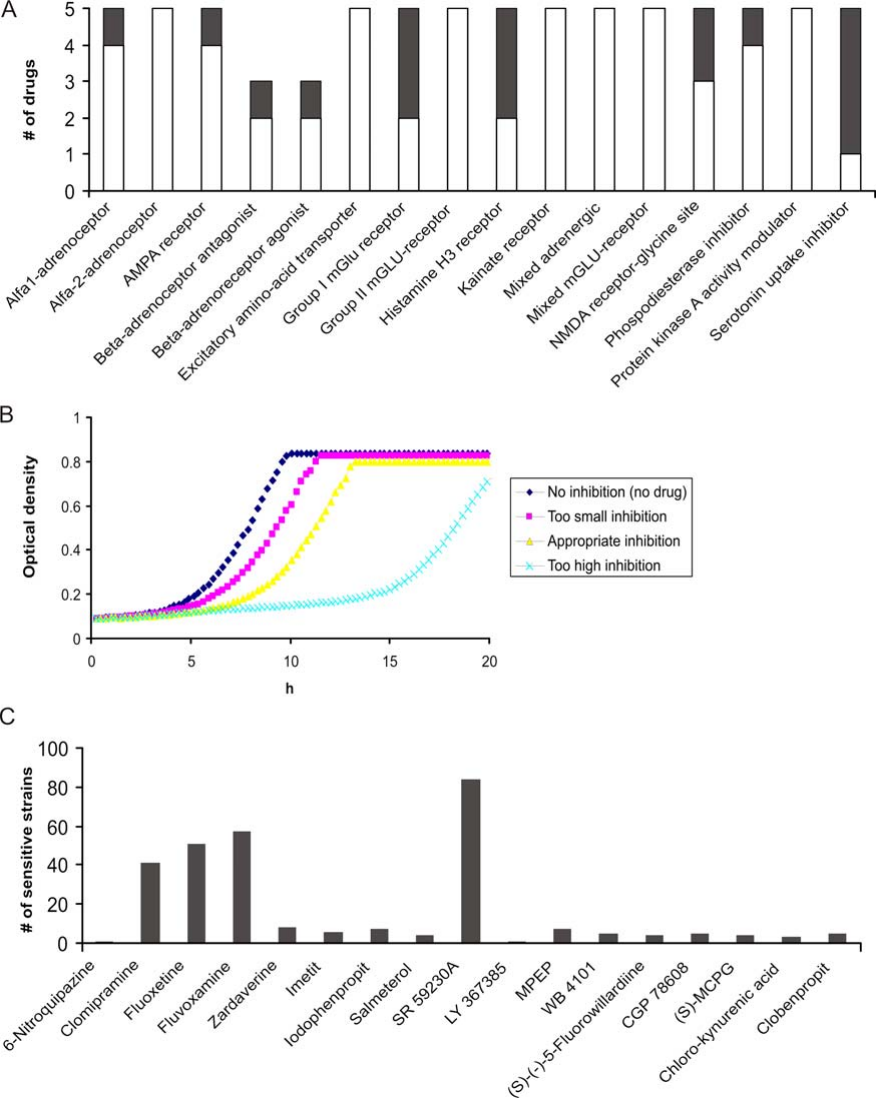
Serotonergic Drugs Showed Potency on Yeast. (A) Number of drugs that did (black) or did not (white) inhibit wildtype yeast growth for each of the initial drug sets tested. (B) Titration of drug concentrations used in genome-wide screening. Wildtype yeast growth in serial dilutions of drug was recorded as optical density every 15 min over a 25 h period. (C) Number of deletion strains that were sensitive (r>2 and z>3, see Materials and Methods) to bioactive drug in genome-wide fitness profiles.

To ask if psychoactive compounds can inhibit wildtype budding yeast growth, we challenged yeast with 76 high-purity psychoactives representing 16 ligand categories that encompass a broad spectrum of treatments for neurological disorders (see Figure 1 for workflow and Table S1 for drug information). Despite the fact that yeast lacks the established neuronal targets of these compounds, 17/76 (22%) drugs inhibited the growth of wildtype yeast (when tested at 200 µM) and are hereafter referred to as “bioactive”. This observation shows that in addition to their reported targets, many of these compounds also have secondary mechanisms of action. In fact, over half of the 16 tested ligand classes included compounds that were bioactive (Figure 2A). Among these, serotonin uptake inhibitors were most effective; four of five tested molecules in this class inhibited yeast growth (Figure 2A). Because our assay depends on growth inhibition in order to observe any effects on specific deletion strains, we proceeded with the 17 bioactive compounds and determined a drug dose that inhibited wildtype growth by ∼15% (Figure 2B, Table S1). In our previous genome-wide studies this level of inhibition best captured the ability to identify the known drug target while minimizing the number of generally sensitive strains [22],[23]. Applying this drug dose, we subjected the bioactive compounds to genome-wide parallel fitness profiling. In this technique, pools of deletion strains are grown competitively for several generations in the presence of a sub-lethal concentration of drug, and genomic DNA is extracted. After PCR-amplification of the unique molecular barcodes incorporated into each gene deletion cassette, the relative role of each gene for growth in the presence of drug is determined by hybridization of the PCR products to a DNA microarray carrying the barcode complements [27]–[29]. The relative abundance of sequence tags in the drug experiments is compared to control experiments and fitness ratios and z-scores are calculated (see Materials and Methods). We used two pools of diploid strains: *i)* heterozygous deletion strains deleted for one copy of the essential genes (1158 strains), which often identifies compound targets through **H**aplo**I**nsufficiency **P**rofiling (**HIP**) [22],[30], and *ii)* homozygous deletion strains deleted for both copies of non-essential genes (4768 strains); this **HO**mozygous **P**rofiling (**HOP**) assay identifies genes that buffer the drug target pathway [24].

Using this combination of the HIP and HOP assays we found that only a few deletion strains (∼5) exhibited significant sensitivity to most of the 17 bioactive compounds (Figure 2C). In contrast, several deletion strains (∼50) were scored as sensitive for the α1-adrenoceptor antagonist SR 59230A and the three selective serotonin re-uptake inhibitors fluoxetine (Prozac), clomipramine, and fluvoxamine (Figure 2C). Given this unexpected potency of the serotonergic drugs in our yeast assays, we extended our investigation to encompass pharmacologically related agents and screened two commercially available drug libraries encompassing 95 serotonergic and 55 dopaminergic compounds. These drug libraries contained the four FDA-approved serotonergics sertraline (Zoloft), fluoxetine (Prozac), paroxetine (Paxil), and cyproheptadine (Periactin), and the four FDA-approved dopaminergics bromocriptine (Parlodel), clozapine (Clozaril), haloperidol (Haldol), and pimozide (Orap). Based on our initial results, we anticipated a high rate of bioactivity on yeast for these two drug classes. Indeed, 66/150 (44%) of the serotonergic and dopaminergic drugs were bioactive, a significant difference compared to the 22% of the initially screened drugs that represented the 16 different ligand sets (p<10^−7^).

### Physiochemical Properties Separating Active from Inactive Drugs

**Figure 3:**
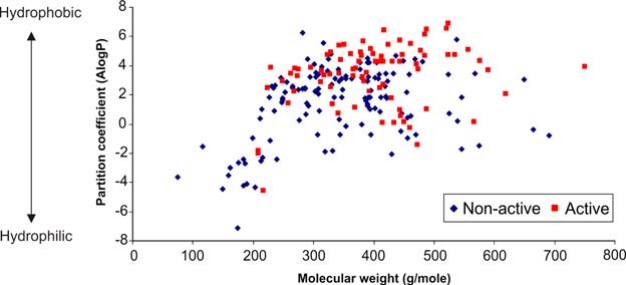
Hydrophobicity and Molecular Weight Discrimination for Non-Active and Active Compounds. All compounds tested were plotted as a function of logP and molecular weight.

The high prevalence of bioactivity in yeast prompted us to ask if any particular psychoactive drug attribute correlated with the ability of these compounds to inhibit wildtype yeast growth. We first performed structural clustering of all ∼220 screened psychoactive compounds using chemical fingerprints in Pipeline Pilot (Accelyrs, San Diego). As more than half of the resulting clusters contained both active and inactive drugs, chemical structure was not predictive of drug action on wildtype yeast growth for this selection of compounds (data not shown). We next asked if any physiochemical properties, as predicted from the structures, were linked to drug activity. The parameters we tested included the number of H-bond donors and acceptors, molecular weight, and hydrophobicity as measured by AlogP (the octanol-water partition coefficient). These measures are important descriptors used in the empirical parameter set known as Lipinski's Rule of Five [31]. In addition to the Lipinski descriptors, we tested six other parameters relevant to drug activity: van der Waals surface area, molecular surface area, molecular solubility, logD (the octanol-water distribution coefficient; a combination of logP and pKa), number of rings and number of rotatable bonds. Principal component analysis revealed that a partition coefficient of AlogP>3 was best able to predict drug activity (p<4.9e-13, for details see Materials and Methods) as shown in Figure 3. A molecular weight of >260g/mole was also indicative of an active compound (p<3.4e-05, Figure 3). If there is a correlation between human side-effects and conserved cellular pathways scored using our surrogate yeast system, it is possible that an additional study could help predict such effects based on structural features.

### Genome-Wide Fitness Profiles on Bioactive Drugs

**Figure 4:**
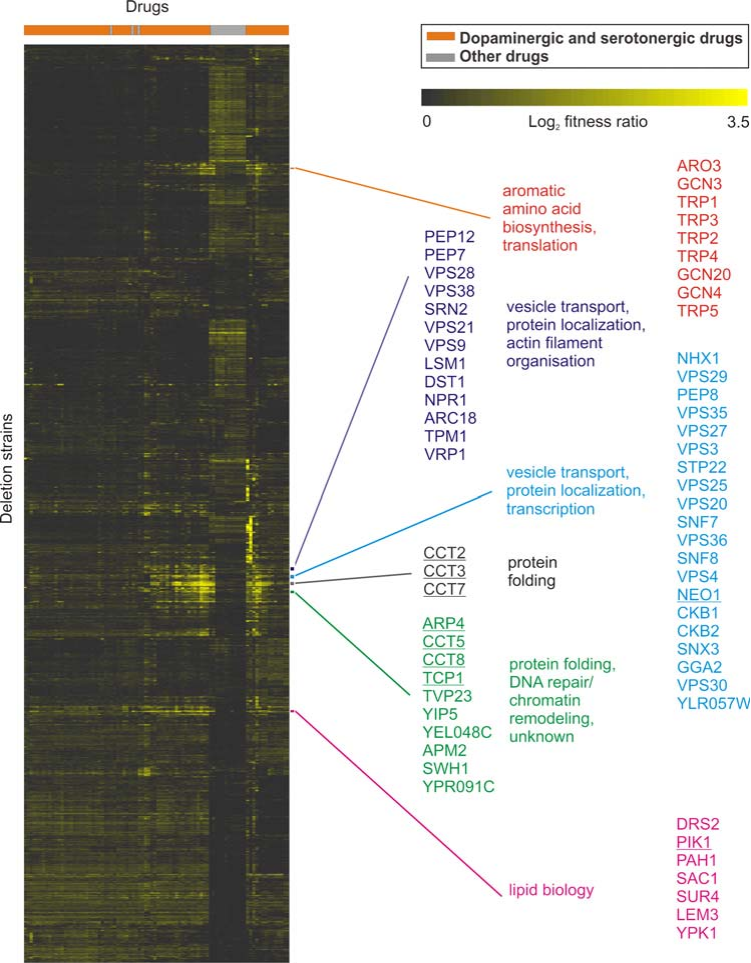
Global Landscape of Fitness Profiles. Two-dimensional hierarchical clustering [32] was used to group all log_2_-fitness ratios obtained from the 81 drugs. Log_2_-fitness ratios from 0 (no fitness defect) to 3.5 (severe phenotype) are color-coded according to the severity of the sensitivity (this paper focuses on sensitivities, see Materials and Methods). Only 0.1% of the log_2_-fitness ratios were higher than 3.5 and became saturated in the figure. The separation of dopaminergic and serotonergic drugs (orange) from drugs in other categories (grey) is indicated. Groups of strains exhibiting highly similar fitness profiles across the psychoactive drugs are extracted from the global clustergram, and the deletion strains included in each group are listed in the order determined by the hierarchical clustering algorithm. For each group of strains, the dominant function(s) of the deleted genes is indicated. Essential genes are underlined.

To systematically interrogate compound mechanisms of action, we subjected the 66 bioactive serotonergic and dopaminergic compounds to genome-wide fitness assays using the approach described above (Figure 1). Combined with the initial set of 17 bioactive drugs, we screened a total of 81 unique drugs (two drugs occurred in duplicate in the chemical libraries), eight of which are used therapeutically (Table 1). Fitness ratios and z-scores for all deletion strains are provided in Tables S2 and S3, respectively (raw data are available at ArrayExpress, EMBL-EBI, accession number E-MTAB-34). The genome-wide fitness profiles were reproducible as the average correlation coefficient for the five replicated compounds was 0.83, which is similar to the average correlation coefficient of 0.72 reported in a previous large-scale fitness study [23]. As an unbiased control, we calculated the average correlation coefficient between all possible random drug pairs in our assay. As expected, this value (0.44) was lower than the average correlation coefficient for duplicates, but well above the previously noted average correlation of zero for unrelated compounds (Maureen Hillenmeyer, unpublished data). In agreement with this, two-dimensional hierarchical clustering [32] did not separate the dopaminergic and serotonergic profiles into two distinct groups, but clearly separated drugs from these two classes from most other compounds profiled (Figure 4). Further indicating the general similarities between dopaminergic and serotonergic drugs in our yeast screen, 25% of the significantly sensitive strains (r>2, z>3, see Materials and Methods) scored in both drug categories (Table S4).

**Table 1:** Examples of Therapeutic Use of the Profiled FDA-Approved Drugs.

Compound	Therapeutic use
Clozapine	Schizophrenia
Cyproheptadine	Schizophrenia, serotonin syndrome, allergy, eating disorder
Fluoxetine	Depression, obsessive compulsive disorder, bulimia nervosa
Paroxetine	Depression, anxiety disorder, obsessive compulsive disorder, panic disorder, post-traumatic stress disorder
Sertraline	Depression, obsessive compulsive disorder, post-traumatic stress disorder
Pimozide	Schizophrenia, psychosis
Haloperidol	Mania, bipolar disorder, schizophrenia, psychosis, nausea, vomiting, restlessness, agitation and aggression, Tourette's syndrome and other tic disorders, hiccups
Bromocriptine	Parkinson's, pituitary tumours, female infertility, overproduction of breast milk

### Core Cellular Processes that Confer Resistance to Psychoactive Compounds

**Figure 5:**
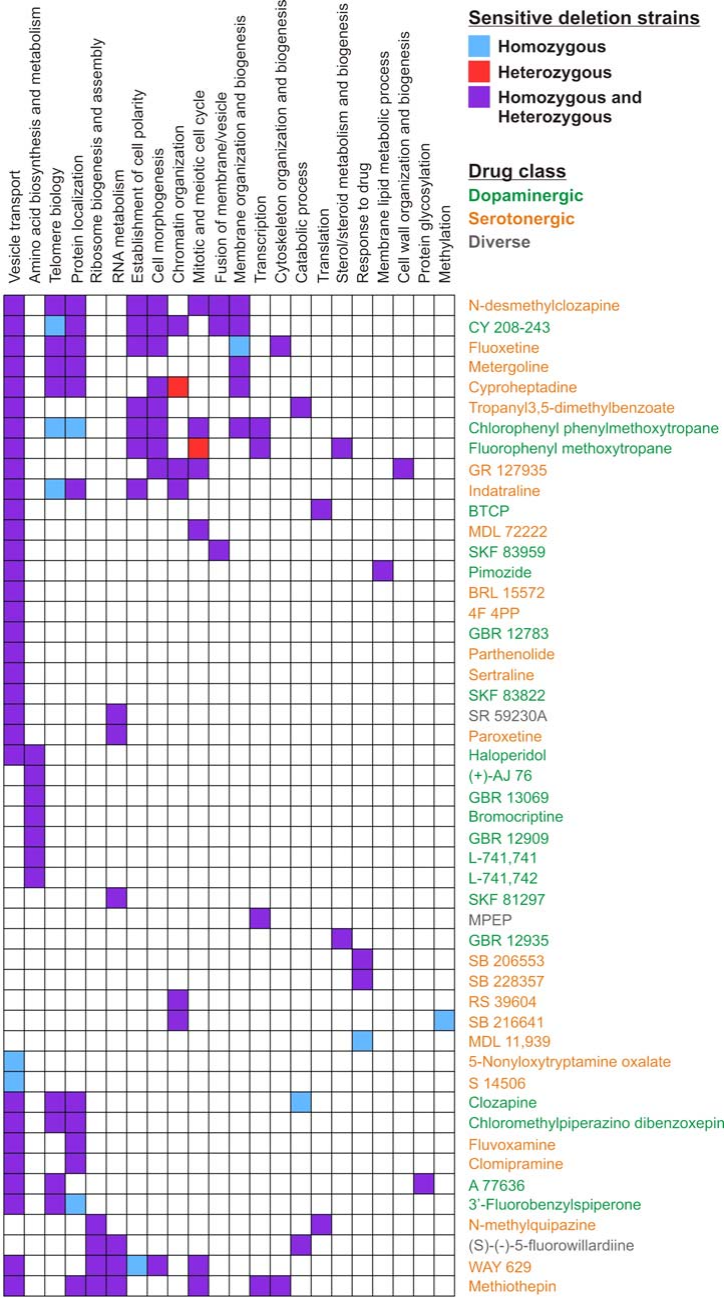
Enriched GO Processes. Significant enrichment (p<0.0001) for GO Processes uniquely scored using sensitive (z>2) homozygous strains (blue) or heterozygous strains deleted for essential genes (red). GO Processes scored using both strain pools are indicated in purple (for details see Materials and Methods). Dopaminergic drugs are indicated in green, serotonergic in orange, and other drugs (from the initially analyzed diverse set) in grey. Drugs with affinity for both a dopaminergic and a serotonergic receptor are indicated according to which Tocris drug library they belong to. Closely related GO categories are collapsed for clarity (see Table S6).

To ask which cellular functions and pathways were required for resistance to the tested drugs, we performed functional enrichment tests using Gene Ontology (GO) annotations specifically focusing on sensitive strains in the *i)* essential heterozygous, *ii)* homozygous or *iii)* both collections (see Materials and Methods). 32 drug sensitivity profiles were not enriched for any GO Process but the remaining 49 profiled drugs (60.5%) interfered with 106 different processes (multiple-testing corrected p-value<0.0001, Table S5). For visual clarity, we collapsed these 106 processes down to 22 (Table S6). The drug sensitivity profiles obtained with the combined set of heterozygous and homozygous strains were enriched for the highest number of condensed GO processes (119 processes, purple color in Figure 5), while 12 processes were uniquely enriched among sensitive homozygous deletion strains (blue color in Figure 5). These processes likely reflect drug detoxification mechanisms (*e.g.* “vesicle transport” and “response to drug”) or other processes required for resistance to compound by an unknown mechanism (*e.g.* “amino acid biosynthesis and metabolism”). Two processes were uniquely scored for essential genes (red color in Figure 5) and are further discussed below.

Investigating the general nature of our enrichment profiles, we found that the most frequently enriched processes across all drugs and genetic backgrounds were vesicle transport, protein localization, and telomere biology (Figure 5). Genes functioning in cell morphogenesis, establishment of cell polarity, cell cycle, amino acid biosynthesis, chromatin organization, RNA metabolism, and membrane organization were also needed for resistance to several (>5) of the psychoactive drugs. A few GO Processes were unique to a single drug: protein glycosylation (A77636), methylation (SB 216641), cell wall organization and biogenesis (GR 127935), and membrane lipid metabolic process (pimozide). In the subsequent sections we focus on the analysis of the FDA-approved drugs and summarize the most notable enrichments for these drugs in Table 2. First, we discuss identified buffering pathways and drug detoxification mechanisms. Next, we concentrate on potential new drug targets identified for the therapeutically used psychoactive drugs.

**Table 2:** Most Notable Off-Target Effects of FDA-Approved Drugs.

Drug	Functional enrichment (p<0.0001)*
Clozapine	Vesicle-mediated transport
Cyproheptadine	*Chromatin-remodelling*
Fluoxetine	*Establishment of cell polarity*
Paroxetine	*RNA processing*
Sertraline	Vesicle-mediated transport
Pimozide	*Membrane lipid metabolic process*
Haloperidol	Aromatic amino acid biosynthesis
Bromocriptine	Aromatic amino acid biosynthesis

*: Italics = unique enrichment among FDA-approved drugs.

### Uncompromised Cellular Transport, Protein Localization, and Telomere Maintenance Is Important for Resistance to Psychoactive Drugs

Vesicle transport was the most commonly overrepresented process among genes required for resistance to psychoactive drugs (Figure 5) suggesting that uncompromised vesicle transport function is a general requirement for psychoactive drug detoxification. The enrichment of cellular transport genes was especially pronounced in response to clozapine treatment, where 9 of the 10 most required genes belonged to this category (Table 3). Protein sorting and localization accounted for the second most frequently enriched process (Figure 5). Deletion of vesicle trafficking and protein localization genes often resulted in very severe phenotypes (bright yellow in Figure 4). Gene products with protein localization roles include those involved in selecting cargo proteins for endosome-to-Golgi retrieval (*e.g.* Vps29), and those involved in sorting proteins in the vacuole (*e.g.* Pep8). Interestingly, the fitness profiles obtained with certain vesicle transport and protein localization deletions clustered with those obtained with strains deleted for genes functioning in actin filament organization/stabilization (*arc18Δ*, *tpm1Δ*, *vrp1Δ*,), mRNA degradation (*lsm1Δ*), and stabilization of membrane amino acid transporters (*npr1Δ*) (Figure 4, left text panel). A second, large group of strains mainly deleted for genes functioning in vesicle transport and protein localization exhibited similar phenotypes across the 81 drugs as *ckb1Δ* and *ckb2Δ*, which are deleted for genes functioning in regulation of transcription and mitotic cell cycle (Figure 4, right panel).

**Table 3:** Top-Ten Sensitive Deletion Strains in Genome-Wide Profiles of FDA-Approved Serotonergic and Dopaminergic Drugs*.

Clozapine		Cypro-heptidine		Fluoxetine		Paroxetine		Sertraline		Pimozide		Halo-peridol	
**NEO1**	V	**NEO1**	V	**NEO1**	V	**NEO1**	V	**NEO1**	V	**MCD4**		**NEO1**	V
PEP7	V	**ARP4**	D	**RPL32**	R	**TOA2**	R	**CCT7**	P	**GSP1**	R	TRP4	A
VPS35	V	PEP12	V	**TCP1**	P	**CFT1**	R	**RPL32**	R	PEP12	V	**ARP4**	D
PEP12	V	**TUB1**	P	VPS30	V	**SEC4**	V	**GSP1**	R	**TUB4**	P	**ERG11**	
SAC1	V	**CCT7**	P	CAP2	P	**CCT8**	P	**NOP8**	R	SEC22	V	ARO2	A
VPS29	V	VPS35	V	PEP12	V	**RPL32**	R	**BET1**	V	PGD1	R	APL2	V
PEP8	V	**CCT3**	P	**CCT3**	P	**FHL1**	R	**ARP4**	D	THP2	R	ARO1	A
VPS20	V	**CCT4**	P	**MRS5**	V	**GSP1**	R	**NUS1**	V	**PIK1**		RCY1	
NHX1		VPS30	V	PEP8	V	**GCD11**	R	**CCT3**	P	YPK1		VPS29	V
LEM3	V	VPS29	V	**DFR1**		**RHO1**		PEP12	V	PEP7	V	**PIK1**	

*: Essential genes are in bold, and gene functions that occur frequently in the table are indicated with letters. V = vesicle transport, protein transport and localization, D = DNA repair, P = protein folding, actin and tubulin assembly, R = RNA processing, transcription, translation, ribosomal function, A = amino acid biosynthesis and metabolism. For bromocriptine, we scored only one significantly sensitive strain, *trp3Δ*, which is involved in aromatic amino acid biosynthesis (z>3, r>2). Strains were sorted according to their fitness ratio, using average ratios for replicated drugs and removing dubious ORFs.

Most of the drug sensitivity profiles were enriched for both protein localization and telomere biology (Figure 5). The apparent “linking” of these enrichments could be attributed to genes that are, in fact, involved in both these processes. Examples of such genes function in the three Endosomal Sorting Complexes Required for Transport, more specifically in ESCRT I *(VPS28, STP22*), in ESCRT II *(SNF8* and *VPS25),* and in ESCRT III *(SNF7*). These genes are, in addition, associated with telomere defects [33],[34].

### The Atypical Antipsychotic Clozapine Has No Fewer Off-Target Effects than Typical Antipsychotics

Because the more recently developed atypical antipsychotic drugs are still associated with side effects and their benefits are currently debated, we compared the phenotypic profiles of the atypical antipsychotic clozapine to two traditional antipsychotics, reasoning that if atypical drugs are more specific, they would exhibit fewer off-target effects in yeast. In contrast to this expectation, the atypical antipsychotic clozapine exhibited a similar number of significantly sensitive (r>2, z>3, see Materials and Methods) deletion strains (26) as the typical antipsychotic drugs pimozide (29) and haloperidol (20). Comparing the fitness profiles of clozapine with the typical antipsychotics pimozide and haloperidol, we found that each drug was associated with unique functional enrichment profiles: clozapine for telomere biology and protein localization, pimozide for membrane lipid metabolic processes, and haloperidol for aromatic amino acid biosynthesis and metabolism (Figure 5). In contrast, vesicle transport was enriched in all three drug sensitivity profiles. The more detailed GO processes behind the condensed process vesicle transport were vesicle-mediated transport for all three drugs and, in addition, secretory pathway, secretion, post-Golgi vesicle-mediated transport and Golgi vesicle transport for haloperidol and clozapine (Tables S5 and S6). The distinct fitness profiles are consistent with the structural differences that exist between these drugs (Figure S1). For example, clozapine has substructures (piperazine and diazepine) that do not exist in pimozide and haloperidol, and haloperidol contains two benzene rings while pimozide has three.

### Bromocriptine, Haloperidol, and Five Additional Dopaminergic Drugs Interfere with Amino Acid Biosynthesis and Metabolism

Compared to the other investigated therapeutics, the fitness profile in the anti-Parkinson drug bromocriptine pointed to a single potential off-target mechanism of action for this drug. The only overrepresented function among sensitive strains was amino acid biosynthesis and metabolism (Figure 5) and the most sensitive strains were deleted for the aromatic biosynthesis genes *TRP3, TRP4*, *TRP1*, *ARO1*, *TRP2*, and *ARO2*. In addition to bromocriptine, six other dopaminergic drugs also interfered with amino acid biosynthesis and metabolism (Figure 5). The sensitivity profiles of all these seven drugs shared the enrichment for the detailed GO process aromatic compound metabolic process (Tables S5 and S6) due to the sensitive phenotype of 13 strains in total. Among them, strains deleted for *TRP1*, *TRP2*, *TRP3*, *TRP4*, *TRP5*, *ARO2*, and *ARO3* were scored in all 7 drugs and strains deleted for *ARO1* and *ARO7* in 6 drugs. Besides the notable enrichment for genes involved in aromatic compound metabolism, the sensitivity of strains missing other genes also contributed to the observed GO process enrichment. Such genes included the folic acid (vitamin B9) biosynthesis gene *FOL2*, the panthothenate (vitamin B5, precursor of coenzyme A) biosynthesis gene *FMS1*, and the protein kinase *GCN2*, which induces amino acid biosynthesis genes in yeast in response to starvation and, in addition, restricts intake of diet lacking essential amino acids in rats [35].

### Pimozide Is Unique in Being Enriched for Membrane Lipid Metabolic Process Genes

The sensitivity profile of the typical antipsychotic pimozide showed a unique enrichment for membrane lipid metabolic processes not seen for any of the other 80 profiled drugs (Figure 5). In pimozide, the *MCD4*-deletion strain had the strongest phenotype and was 21-fold depleted compared to the control (Table 3). *MCD4* is highly conserved among eukaryotes and functions in glycosyl-phosphatidylinositol (GPI) anchor synthesis. Because *MCD4* is an essential gene, it may represent an additional, clinically relevant drug target for pimozide. The inositol-lipid-mediated signaling gene *PIK1* and the spingholipid-mediated signaling gene *YPK1* were also among the ten most required genes for resistance to pimozide (Table 3). They clustered with a group of other strains deleted for genes involved in lipid biology (Figure 4), such as the *de novo* lipid synthesis genes *PAH1* and *SUR4*.

### Fluoxetine Interferes with the Establishment of Cell Polarity

Eight drugs, among them the antidepressant fluoxetine, were enriched for the condensed term establishment of cell polarity (purple or blue color in Figure 5). In total, 51 genes were assigned to the detailed GO process establishment and/or maintenance of cell polarity and caused a sensitive phenotype when deleted (Tables S5 and S6). Many of these genes scored in the majority of the drugs, for example all four members (*CKA1*, *CKA2, CKB1*, and *CKB2*) of the casein kinase II-holoenzyme complex, and *TPM1*, the major isoform of tropomyosin which directs polarized cell growth and organelle distribution. For the seven drugs where the enrichment for establishment and/or maintenance of cell polarity was scored using sensitive homozygous and essential heterozygous strains (purple color in Figure 5), six essential members (*EXO70*, *SEC3*, *SEC6*, *SEC8*, *SEC10* and *SEC15*) of the exocyst complex, which determines where secretory vesicles dock and fuse, were scored in all drugs except fluoxetine.

### Essential Genes of High Importance for Drug Resistance May Reveal Additional Secondary Drug Targets for Psychoactive Drugs

Drug targets are often encoded by essential genes, thus essential genes scored in our assay may represent important additional targets of psychoactive compounds that may be useful in the development of therapeutics for other applications. In a given heterozygous strain, the reduced gene copy number of a potential drug target leads to a reduced level of the corresponding protein. When this strain is grown in the presence of a drug targeting the heterozygous locus, the result is a further decrease in “functional” dosage due to the drug binding to the protein target. If this protein is important for growth, the result will be drug sensitivity [22]. In our functional enrichment tests, two processes were uniquely overrepresented among sensitive essential genes (red color in Figure 5): mitotic and meiotic cell cycle for fluorophenyl-methoxytropane and chromatin organization for cyproheptadine. Examples of targeted essential genes in cyproheptadine treatment include chromatin-remodeling genes (*ARP4*, *ARP7*, *ARP9*), genes in the multisubunit (NuA4) histone acetyltransferase complex (*EPL1*, *ESA1*, *SWC4*), and *RSC4* and *RSC6* in the RSC Chromatin remodeling complex.

Although not revealed as a functional enrichment among sensitive strains deleted for essential genes, most of the other FDA-approved drugs also have potential secondary drug targets as infered by the presence of essential genes among the ten most required genes for drug resistance (Table 3). As judged by the high number of sensitive strains deleted for essential genes in paroxetine treatment (10 strains) and sertraline treatment (9 strains), these selective serotonin re-uptake inhibitors are particularly rich in potential secondary drug targets. Essential genes required for resistance to the FDA-approved drugs include those involved in RNA processing, transcription and translation, genes functioning in the protein folding chaperonin complex, and the chromatin-remodeling/DNA repair gene *ARP4* (bold in Table 3). Deletion of *ARP4* resulted in some of the most sensitive phenotypes when cells were treated with cyproheptadine, sertraline, or with haloperidol (Table 3). *ARP4* has a close human homolog, *ACTL6B*, which encodes a subunit of the BAF (BRG1/brm-associated factor) complex in mammals, functionally related to the SWI/SNF complex in *S. cerevisiae*. The SWI/SNF complex is thought to facilitate transcriptional activation by antagonizing chromatin-mediated transcriptional repression [36]. Another example of an essential gene required for drug resistance in several FDA-approved drugs is *GSP1*, which functions in RNA-processing (Table 3). The mammalian homolog of Gsp1, Ran (BlastP E-value<E-261) is, as in yeast, a nuclear GTP-binding protein.

Interestingly, the fitness profile of the *ARP4*-deleted strain was very similar to the strains deleted for the cytosolic chaperonin subunits *CCT5*, *CCT8* and *TCP1* (Figure 4). The chaperonin complex is involved in protein folding (primarily of actin and tubulin) and cytoskeleton organization [37]. In our fitness assays, seven of eight CCT-strains scored as significantly sensitive in many of the probed psychoactive drugs. Some (*CCT3, CCT4, CCT7* and *CCT8*) were even among the top-ten required genes for resistance to cyproheptadine, fluoxetine, paroxetine, and sertraline (Table 3). Furthermore, several deletion strains with uncharacterized functions had similar fitness profiles as the chaperonins *CCT5*, *CCT8* and *TCP1* (Figure 4). Among them were *TVP23* and *YIP5* which both localize to the late Golgi, *YEL048C* which is synthetic lethal with *GCS1* (involved in ER to Golgi transport), *APM2* (homologous to medium chain of mammalian clathrin-associated protein complex involved in vesicle transport) and *SWH1* (similar to mammalian oxysterol-binding protein, localized to Golgi and nucleus-vacuole junction).

### Psychoactive Drugs often Impinge on Evolutionarily Conserved Processes

To test if our findings in yeast might reflect drug action in human cells, we looked at the proportion of scored genes with human homologs. Among the strains significantly sensitive to at least one psychoactive compound, 58.4% were deleted for a gene with a close human homolog (BlastP E-value<E-6), as compared to 45.0% for all analyzed deletion mutants regardless of whether they had a fitness defect or not. To test if strains deleted for genes involved in core cellular processes are more sensitive in general, we compared our results obtained with the 81 psychoactive compounds to 81 randomly chosen chemically diverse compounds (see Materials and Methods). We found that a similar proportion of genes with close human homologs (59.7%) were scored for strains that were significantly sensitive to at least one of these diverse chemicals. Despite this similarity in proportion of sensitive strains with human homologs in the two datasets, conserved genes were scored much more frequently (in >10% of the compounds) in the psychoactive drug set than in the random drug set. In fact, considering only genes deleted in frequently scored strains, 64.1% of the psychoactive drugs had close human homologs (BlastP E-value<E-6) while the corresponding proportion for the structurally diverse drug set was significantly (p<0.006) lower (45.4%) and similar as the fraction of human homologs for multi-drug resistance genes (47.1%) in a recently published study [23]. This difference points to a significant enrichment of frequently scored sensitive strains with human homologs for the psychoactive drugs. Among the strains sensitive to the highest number of psychoactive compounds, seven of eight had close human homologs: *NEO1*, *SAC1*, *PIK1*, *VPS29*, *PEP8*, *ARP4* and *VPS35*. The majority of these genes are involved in vesicle transport, which was the most frequently enriched function among strains sensitive to psychoactive drugs. Thus, the specific psychoactive drug detoxification mechanisms identified in yeast are likely to be of importance in humans treated with psychoactives.

## Discussion

Many psychoactive drugs are associated with adverse secondary effects in humans yet the mechanisms that underlie these off-target effects are poorly understood. To address mechanisms of drug action in a systematic manner, we profiled the genome-wide collection of budding yeast deletion strains for sensitivity to a broad spectrum of psychoactive compounds, of which dopaminergic and serotonergic drugs had a high bioactivity. Among 214 tested compounds, we uncovered 81 drugs that conferred a measurable growth defect on wildtype yeast. An appropriate dose of these active compounds was applied to the pooled heterozygous and homozygous yeast deletion sets to identify genes whose function is required for optimal growth in the presence of drug. Fifteen percent of all yeast strains (deleted for non-dubious ORFs) exhibited significant sensitivity (r>2, z>3) to these 81 psychoactive compounds and more than half of the drugs interacted with core cellular functions. Several clinically important drugs, such as fluoxetine, cyproheptadine, and clozapine were linked to diverse cellular processes. This observation may explain both the diversity of side effects observed in human patients and the therapeutic variability associated with these drugs. That is, polymorphisms in any of the conserved processes affected by a given drug are a likely source of the individual variation in response to drug. For instance, the response to the frequently prescribed antipsychotic clozapine is highly variable between individuals as the same dose can have markedly different efficacy and/or side effects in different patients [38]. Genes functioning in vesicle transport, protein localization, telomere biology, and catabolic processes were required for clozapine resistance in yeast. In another example, fluoxetine is associated with side effects such as seizures, nausea, sleepiness, anxiety, and serious allergic reactions. This antidepressant affects numerous cellular processes including establishment of cell polarity, protein localization, and cytoskeleton organization and biogenesis. Given the limited number of FDA-approved drugs within the set of 81 compounds analyzed here and the overlapping side effects associated with these drugs, it is not yet possible to correlate any single side effect to a particular perturbed pathway.

The most frequently scored sensitivity for the 81 profiled antipsychotic drugs was due to loss of secretory pathway function, likely indicating the importance of vesicle transport (*e.g.* to the vacuole) for drug detoxification. The lysosome (the mammalian vacuole equivalent) is known as the major site of degradation of both exogenous and endogenous molecules. For FDA-approved drugs, the requirement for vesicle transport genes was reflected in the frequent sensitivity of the *neo1* deletion strain as the most sensitive strain in six FDA-approved drugs. Neo1 is an essential, highly conserved type 4 P-type ATPase involved in intracellular membrane- and protein-trafficking. Members of this family of P-type ATPases are implicated in the translocation of phospholipids from the outer to the inner leaflet of membrane bilayers. Our data suggested that interference with membrane structure and transport through inhibition of Neo1 is an additional, unwanted mechanism of action for clozapine, cyproheptadine, fluoxetine, paroxetine, sertraline and haloperidol, and their drug analogs. The importance in humans of functional 4 P-type ATPases is well documented as hereditary cholestasis, caused by defects in biliary epithelial transporters, has been directly linked to mutations in a 4 P-type ATPase gene [39].

In addition to the frequently observed requirement for uncompromised vesicle transport for drug detoxification, several drug sensitivity profiles were enriched for more specific processes. Within the FDA-approved drug group, the antidepressant paroxetine was unique in targeting RNA processing genes, pimozide interfered with membrane lipid metabolic processes, cyproheptadine preferentially targeted essential genes with chromatin remodelling functions, and fluoxetine interfered with establishment of cell polarity. Furthermore, seven dopaminergic compounds including the anti-Parkinson drug bromocriptine resulted in sensitivity of strains deleted in aromatic amino acid biosynthetic genes. This sensitivity may be a result of that dopaminergic drugs block aromatic amino acid uptake in yeast, requiring yeast to activate the corresponding biosynthetic pathways. Given the fact that aromatic amino acids are precursors to dopamine and serotonin, this was an interesting observation suggesting that the levels of intracellular precursors may be important in the response to certain psychoactive drugs.

Interestingly, interference with members of the chaperonin complex resulted in some of the most severe phenotypes. Seven of eight CCT-strains scored as significantly sensitive in several psychoactive drugs, among them *CCT5*. The human homolog of this gene is associated with hereditary neuropathy [40]. Although it is unclear how mutated *CCT5* causes this disease, it has been postulated that its mutation leads to accumulation of misfolded cytoskeletal proteins, leading to defective assembly of actin into microfilaments resulting in neuronal apoptosis [41]. In our yeast screens, *CCT5* was needed for resistance to eight different compounds (cyproheptadine, paroxetine, fluoxetine, indatraline, MDL72222, CY208-243, 2-Chloro-11-(4-methylpiperazino)-dibenz[b,f]oxepin, N-Desmethyl-clozapine, and 3-alpha-[(4-Chlorophenyl)-phenylmethoxy]-tropane. We conclude that interference with tubulin and actin folding is an important, secondary mechanism of action of these compounds.

As an example of how the information from our yeast assays may lead to testable drug-gene interaction hypotheses in humans, we found that the levels of the yeast strain heterozygous for *ACC1* was eleven-fold reduced in ritanserin as compared to the control, indicating that the acetyl-CoA carboxylase Acc1 may be a secondary target of ritanserin. Like its yeast counterpart, the human homolog *ACACA* is required for *de novo* biosynthesis of long-chain fatty acids and its activity drops during fasting [42]. Because increased appetite is a reported side-effect during ritanserin treatment [43], it is tempting to speculate that biochemically mimicking fasting would increase appetite.

These studies raise several important issues for further consideration. Understanding the mechanisms that underlie adverse effects of clinically approved drugs is crucial for the development of next generation therapeutics with improved selectivity and efficacy. Moreover, knowledge of patient polymorphisms in off-target pathways may allow adverse effects of any given drug to be preempted by personalized pharmacogenomic strategies. It is also conceivable that some of the observed secondary drug effects are critical for therapeutic benefit.

In summary, a number of cellular processes were associated with sensitivity to the dopaminergic and serotonergic classes of psychoactive compounds. This points to additional, previously uncharacterized mechanisms of action for these drugs in humans and suggests follow-up experiments aimed at understanding a drug's mechanism of action on a genome-wide level. Our results suggest that model organism pharmacogenetics can be used as a comprehensive and unbiased tool in initial studies aiming at unraveling secondary effects and mechanisms of action for therapeutic compounds and their analogs. A more rigorous understanding of the complete mechanism of drug action in humans would be beneficial in the development of a new generation of better tolerated psychoactive drugs, and in personalized medicine.

## Materials and Methods

### Compound Libraries

High purity compounds for genome-wide fitness profiles were obtained from Tocris BioScience (http://www.tocris.com) as ligand sets and as the serotonergic (#1732) and dopaminergic (#1718) collections. In total, these drug collections comprised 226 drugs, 12 of which overlapped between the collections. A complete list of drugs, catalogue numbers, solvents, and concentration used in the genome-wide screens is provided in Table S1.

### Genome-Wide Yeast Growth Assay

For genome-wide fitness profiles, the complete sets of ∼4700 homozygous deletion strains and ∼1100 essential heterozygous deletion strains in the BY4743 and BY4744 backgrounds (*MATa/α his3Δ1/his3Δ1 leu2Δ0/leu2Δ0 lys2Δ0/LYS2 MET15/met15Δ0 ura3Δ0 /ura3Δ0 ORF::kanMX4*) were used [29],[44]. A strain in the same genetic background with *YDL227C* replaced by *kanMX4* was used as the wildtype control for drug titration curves. Strains were stored in 7% DMSO at -80°C. Because all experiments were performed in rich media (YPD [45], without antibiotics), it is unlikely that the presence of auxotrophies had a major effect on our results, however, we cannot rule out that the disruption of the corresponding pathways in yeast may, in some cases, alter our findings. Beginning from an initial maximal concentration of 200 µM, the degree of growth inhibition was determined by exposing wildtype cells to a serial dilution of compound until only a slight inhibition (∼15%) of wildtype growth was observed (see Figure S1). Cells were inoculated at an OD_600_ of 0.0625 in serial dilutions of drug and grown in a Tecan GENios microplate reader (Tecan Systems Inc., San Jose, USA) at 30°C with orbital shaking. Optical density measurements (OD_600_) were taken every 15 minutes until the cultures were saturated, and doubling time *(D)* was calculated as described [46]. Fitness assays using pooled deletion strains were performed as described [47] with the following modifications: *i)* after growth, 350 µl from each of two independent cultures of the 5-generation homozygous pool and 350 µl from the 20-generation heterozygous essential pool were combined, thereby allowing for approximately equal representation of barcodes for PCR reactions and hybridization to the same DNA chip using the unique barcodes incorporated in each of these strains. *ii)* for amplification of the tags, ∼0.2 µg genomic DNA was combined with a 1 µM mix of either up- or down-tags and 82% (v/v) Platinum High Fidelity PCR Supermix (Invitrogen # 11306-016) containing anti-*Taq* DNA polymerase antibody, *Pyrococcus* species GB-D thermostable polymerase, recombinant *Taq* DNA polymerase, Mg^2+^, and dNTPs, *iii)* extension temperature was 68°, *iv)* extension was for 2 min except for a final 10 min extension *v)* 34 cycles of amplification were performed, *vi)* after 10-16 h, the hybridization mix was removed from Affymetrix Gene Chips, replaced with Wash A (6x SSPE, 0.01% Tween), and chips were stained and washed using GeneChip Fluidics Station 450 (Affymetrix) according to the GeneFlex_Sv3_450 protocol with one additional wash A cycle before the staining.

### Data Analysis

Intensity values for the probes on the chip were extracted using the GeneChip Operating Software (Affymetrix). Quantile normalization, outlier omission, fitness defect ratio (denoted as “r”) and z-score (denoted as “z”) calculations were performed as previously described [47],[48]. In short, fitness defect ratios were calculated for each deletion strain as the log2 of the ratio between the mean signal intensities of the control and the drug chips. The larger the ratio, the more depleted (sensitive) is the strain as compared to control condition without the drug. To include the variance in the control experiments, we also calculated z-scores for each gene by dividing the difference in mean intensity across the control chips and treatment with the mean standard deviation of the signal intensities for the given gene across all 18 control chips [48]. The larger the z-score, the more likely it is that a given strain is significantly depleted from the pool. In our analysis, we scored a deletion strain as significantly sensitive using a threshold for both z-score and log2 intensity ratio. A threshold of z>3 was selected based on our earlier observations that above this limit, 100% of 186 deletion strains detected as sensitive by microarray could be confirmed using individual growth assays [24]. This stringent threshold was chosen to minimize the number of false positives. In addition, we added a further requirement that a sensitive strain should display at least a fourfold depletion (r>2, *i.e.* log2>2) compared to the control condition. This criterion was added to avoid including z-scores which were artificially high due to a low standard deviation in the control chips. Due to the way the screens were performed (at low drug concentration, *i.e.* an IC_15_) and analyzed [22],[24] we have focused on sensitive strains in this work, as opposed to apparently resistant strains. Two-dimensional hierarchical clustering of the fitness ratios was performed using Pearson correlation [32] and the data was visualised using the MultiExperiment Viewer from the TM4 microarray software suite (http://www.tm4.org/index.html).

In each of the 81 profiled drugs, sensitive deletion strains were tested for Gene Ontology Functional enrichment using the standard hypergeometric test provided by the GoStats Bioconductor modules for R [49]. For each drug, we performed three independent functional enrichment tests using *i)* sensitive heterozygous strains deleted for essential genes (z>2), *ii)* sensitive homozygous strains (z>2), and *iii)* all sensitive strains in the given drug with z>2. As the global control set, we used all yeast ORFs in the corresponding deletion background with chip intensity values above background. The background was determined as the average value of all unused tags on the chip (∼3600 tags×5 copies = 18000 values) **+**2 standard deviations of the background tags. Obtained p-values were corrected for multiple testing by multiplying by the number of identified terms. Adjusted p-values<0.0001 were considered significant. GO processes linked to less than 20 or more than 300 genes in our background set were excluded from our tests. Two-dimensional hierarchical clustering of overrepresented GO processes was performed using binary data [50]. To test the robustness of our functional enrichment tests, we repeated the same analysis using each of the following thresholds: z>3, r>2, r>3 and found consistent functional enrichment profiles.

In the calculations of the proportion sensitive strains deleted for genes with close human homologs (Blastp E-value<E-6), we used a set of 81 recently profiled (our unpublished data) compounds with potency against wildtype yeast. These compounds represent structurally diverse chemicals derived from Chemical Diversity Labs, Inc. repository of >500,000 compounds.

Structure data files were obtained from Tocris and Pubchem for all compounds and Babel canonical smile strings were generated. In the chemical structure clustering, extended connectivity fingerprints based on functional classes in Pipeline Pilot were used [51]. In the physiochemical property clustering, ten descriptors representing important properties for potential drug candidates were calculated after salts were stripped, using Frowns and Openeye cheminformatic libraries [31],[52]. PCA was used to find the strongest properties that separated active from non-active compounds. The revealed properties ALogP and molecular weight were validated to see how they correlated with the pattern of the other eight descriptor loadings. The non parametric Wilcoxon rank sum test supported LogP (p-value 4.91e-13) and MW (p-value 3.42e-05) as significant representative properties.

All supplementary data can also be downloaded from our webpage, http://chemogenomics.med.utoronto.ca/Supplemental/psychoactives/.

## Supporting Information

Figure S1 Chemical structures of the atypical antipsychotic clozapine and the typical antipsychotics haloperidol and pimozide.
(0.80 MB TIF)

Table S1 Drugs used in genome-wide fitness profiles. Catalogue number (Tocris), solvent, highest drug concentration tested in wildtype yeast, drug concentration used, and brand names for FDA-approved drugs.
(0.04 MB XLS)

Table S2 Fitness ratios for indicated compounds for all deletion strains with intensity values above background.
(9.73 MB XLS)

Table S3 Z-scores for the indicated compounds for all deletion strains with intensity values above background.
(9.73 MB XLS)

Table S4 Strains scored as significantly sensitive with at least one dopaminergic or one serotonergic drug. For each strain, the number of dopaminergic and serotonergic drugs that caused significant depletion in the pool is shown (r>2, z>3). Strains scored in both drug classes are indicated with "1".
(0.33 MB XLS)

Table S5 Significantly enriched (p<0.0001) GO Processes in genome-wide fitness profiles.
(0.12 MB XLS)

Table S6 Sub-grouping of the enriched GO categories.
(0.02 MB XLS)
