# Supplementary figures and images for: Off-Target Effects of Psychoactive Drugs Revealed by Genome-Wide Assays in Yeast
Source: PLoS Genet. 2008 Aug 8;4(8):e1000151. doi: 10.1371/journal.pgen.1000151 (PMC2483942; doi:10.1371/journal.pgen.1000151)

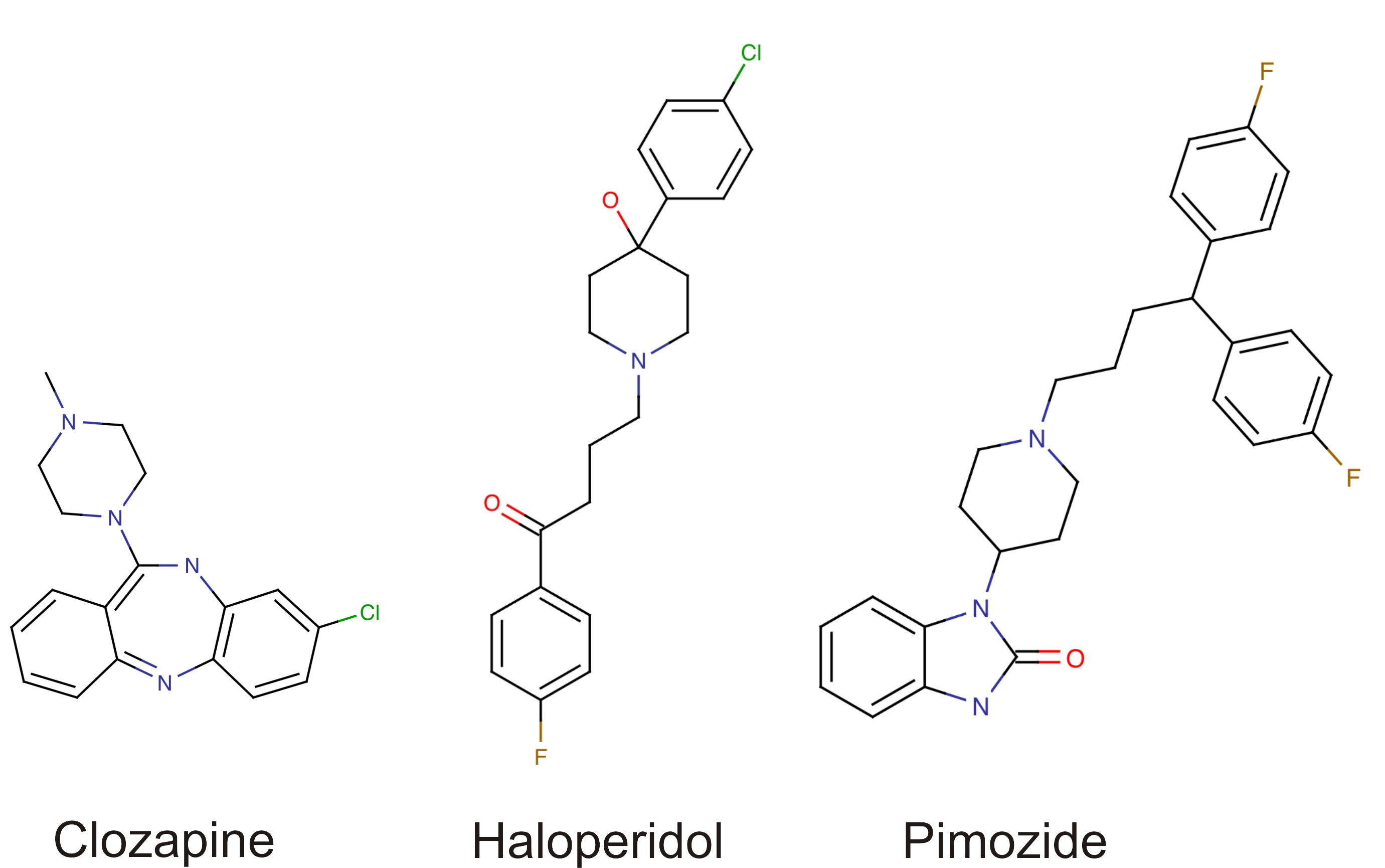

Supplement: Figure S1 — Chemical structures of the atypical antipsychotic clozapine and the typical antipsychotics haloperidol and pimozide. (0.80 MB TIF) [file pgen.1000151.s001.tif]
